# Therapeutic antibodies for infectious diseases

**DOI:** 10.2471/BLT.16.178061

**Published:** 2017-02-02

**Authors:** Erin Sparrow, Martin Friede, Mohamud Sheikh, Siranda Torvaldsen

**Affiliations:** a School of Public Health and Community Medicine, University of New South Wales, Samuels Avenue, Kensington, Sydney, NSW 2033, Australia.; b Initiative for Vaccine Research, World Health Organization, Geneva, Switzerland.; c Sydney Medical School Northern, University of Sydney, Sydney, Australia.

Passive immunization is the transfer of antibodies and occurs naturally during pregnancy. The transplacental transfer of maternal antibodies to the fetus can protect the infant from many infectious diseases for the first vulnerable months of its life. Passive immunization has been used in the global effort to eliminate maternal and neonatal tetanus. Researchers have estimated that vaccinating pregnant women with two or more doses of tetanus-containing vaccine has reduced neonatal mortality from tetanus by 94%.^1^

In addition, clinicians have used passive immunization, to prevent or to treat various infections for over a century for diseases such as rabies, diphtheria, tetanus, hepatitis B, respiratory syncytial virus and botulism. Passive immunization is also used in immunocompromised individuals and to manage complications after vaccination.

Most of the antibody preparations administered to patients, containing polyclonal antibodies, have been derived from sera of immunized animals, immunized humans, and for some rarer diseases, from sera of convalescent patients.^2^^–^^4^ Box 1 presents blood-derived polyclonal antibodies currently in use.^5^^–^^7^ The production and use of polyclonal antibodies have revealed several challenges, including standardization, patient safety, supply and access. These challenges have led researchers to explore the possibility of replacing polyclonal antibodies with monoclonal antibodies (mAbs), which can be produced through recombinant deoxyribonucleic acid technologies.

Box 1 **Indications for blood-derived antibodies for infectious diseases with a current American or European Union market authorization^a^**

- Anthrax: treatment of inhaled anthrax.
- Botulism: treatment of botulinum.
- *Clostridium botulinum*: treatment of infant botulism caused by type A or B *C botulinum* in patients < 1 year.
- Cytomegalovirus: prophylaxis of cytomegalovirus disease associated with transplantation of kidney, lung, liver, pancreas and heart.
- Diphtheria: treatment of diphtheria and rarely as prophylactic of diphtheria in asymptomatic, non-immunized individuals who have been exposed.
- Hepatitis A: protection from hepatitis A in household and other close contacts.
- Hepatitis B: prevention of Hepatitis B recurrence following liver transplantation; treatment of acute exposure to Hepatitis B-containing blood, sexual exposure to infected persons, infants born to infected mothers and household exposure to persons with acute infection.
- Hepatitis C: Prevention of recurrent hepatitis C virus-induced liver disease in liver transplant recipients.
- Measles: postexposure prophylaxis for suspected measles in unvaccinated persons.
- Rabies: postexposure prophylaxis to rabies category III exposure (i.e. single or multiple transdermal bites or scratches, contamination of mucous membrane with saliva from licks; exposure to bat bites or scratches).
- Rubella: prophylaxis of rubella to exposed individuals in early pregnancy.
- *Staphylococcus aureus*: treatment of *S aureus* bacteraemia.
- Tetanus: immediate prophylaxis after tetanus prone injuries in patients not adequately vaccinated, with unknown immunization status, severe deficiency in antibody production or vaccinated patients with high risk wounds.
- Vaccinia: prevention or treatment of vaccinia/smallpox. Treatment and/or modification of conditions which are complications resulting from smallpox vaccination.
- Varicella: prophylaxis against varicella zoster virus infection in at-risk exposed patients.

**^a^** Excluding polyclonal antibodies indicated for immunodeficiency, Rhesus iso-immunization and antivenoms.Source: US Food and Drug Administration^5^^,^^6^ and European Medicines Agency.^7^

In the past, producing mAbs was difficult and expensive. However, the increasing use of mAbs as therapeutics for cancer, autoimmune diseases and other chronic diseases has led to increased production capacities and improved manufacturing processes.^8^ These advancements have made mAbs potentially cost-competitive with blood-derived polyclonal antibodies, while at the same time contributing towards an improved supply at a global level. Over 40 therapeutic mAbs are currently in use, targeting a range of noncommunicable diseases.

In addition to potentially addressing the patient safety and supply limitations of polyclonal antibodies, anti-infective mAbs may offer prophylactic or therapeutic interventions for infectious diseases where drugs or vaccines are not available or are poorly efficacious. For example, the use of broadly cross-reactive mAbs to prevent influenza or neutralizing mAbs against Ebola and Middle East respiratory syndrome coronavirus (MERS-CoV). Furthermore, anti-infective mAbs could be used to target multidrug resistant pathogens, such as *Staphylococcus aureus*, to reduce the incidence of hospital-acquired infections and may help to prevent the emergence of antimicrobial resistance.

Although passive immunization produces a short-lived protection against infections, mAbs have some advantages over vaccines for active immunization. First, protection from vaccination takes longer and often requires several doses to elicit a protective immune response, whereas passive immunization provides a much quicker protection. Second, passive immunization can provide protection in immunosuppressed individuals, who are often at a high risk of acquiring infections. Third, since the mAb manufacturing process is generic, a manufacturer can start production of mAbs with minimal lead-time. Therefore, mAbs could become available faster than vaccines during an emergency.

The 2013–2016 Ebola virus disease outbreak in West Africa demonstrated the need for mAb research and development. In August 2014, two American health-care workers infected with Ebola were treated with Zmapp™, an experimental drug containing three mAbs against Ebola.^9^ Unfortunately, the drug supply was only sufficient to treat seven patients and efforts had to be made to increase production before clinical evaluation could take place.^10^ Efficacy trials in Ebola infected patients in West Africa were completed in January 2016. However, due to the waning epidemic, the trial could only enroll 72 patients out of the planned 200. The outcomes showed that out of the 36 patients who received Zmapp™, eight died, which was less than in the group receiving only standard care (13 of 35 patients died). However, the trial sample size was too low to draw any conclusions.^11^ Despite this setback, anti-Ebola mAb development – which has been ongoing for several years and the first neutralizing mAb reported in 1999^12^ – continues towards the goal of licensure under alternative pathways to standard randomized controlled clinical trials.

While there are numerous mAb products under development for a range of infectious diseases, currently only three have been licensed: palivizumab for prevention of respiratory syncytial virus in high-risk infants; and raxibacumab and obiltoxaximab for prophylaxis and treatment of anthrax.

Numerous mAbs have shown the potential to treat infectious diseases in preclinical evaluation, both in vitro and in animal models. However, not many of these mAbs have reached clinical trials.^13^^–^^16^ A review of the WHO International Clinical Trials Registry Platform^17^ and ClinicalTrials.gov, found that as of November 2016, there were at least 38 mAb products in active clinical development for 14 infectious diseases: Anthrax, *Clostridium botulinum* (botulinum neurotoxin A), *C difficile*, Ebola virus disease, hepatitis B, hepatitis C, Hendra virus, herpes simplex virus, human immunodeficiency virus, influenza, rabies, respiratory syncytial virus, *S aureus* and *S epidermidis*.

While mAbs have great potential to address health threats, their development, approval and use face numerous challenges. In November 2014, the World Health Organization (WHO), in consultation with key stakeholders, mapped scientific and regulatory challenges and requirements for mAbs. Here we present some of the key challenges identified.

First, for rare or emerging diseases, such as Ebola, pandemic influenza or MERS-CoV, demonstrating proof of concept in clinical trials is difficult. The small number of patients, the unpredictable outbreaks and epidemiology and the high fatality associated with the ethical challenges of conducting randomized clinical trials, are obstacles when evaluating mAbs as a treatment for such diseases. Therefore, alternative regulatory pathways for product licensure are needed. However, many countries do not have mechanisms in place for such pathways and there is no framework in place for testing, licensure and use of mAbs. In addition, consensus on acceptable clinical endpoints and definition of conditions under which mAbs would be used, are lacking. Therefore, we need disease-specific well-characterized animal models to demonstrate proof of concept and even efficacy for these diseases.

Second, for fatal diseases, such as rabies, where highly effective polyclonal antibodies are available, but short in supply, conducting randomized controlled trials present ethical and logistical challenges. Therefore, researchers need alternative study designs to evaluate mAbs against such diseases. Furthermore, polyclonal antibodies are conceived to neutralize more virus strains than mAbs. Researchers need to address the breadth of protection of these mAbs. Using in vitro neutralization methods and animal models with a broad number of viral isolates could help provide reassurance of the breadth of protection given by mAbs compared to polyclonal antibodies.

Third, when several mAbs are under development against the same disease, but with different product profiles, such as affinity, protective dose and route of administration, health agencies and donors might have difficulty selecting which product(s) to fund. Furthermore, there exists a challenge when comparing mAbs products due to the lack of international reference preparations.

Fourth, high costs may limit access, especially to those in low-resource settings. Although the production costs of mAbs have been reduced over the last decade, the cost is still high (about 100 United States dollars per gram),^8^ especially if several grams are needed for treatment. The number of grams required will differ greatly depending on the target pathogen. Methods to decrease the cost include enabling lower doses by increasing the affinity of the mAbs or changing the production system to increase yields and/or decrease the cost of goods. Targeted use in high-risk individuals may present a cost-effective strategy.

Fifth, for several disease targets, investors and people working with product development need clarity on whether public health agencies will procure and use the new therapeutics or postexposure prophylactics. Without a known market, biotechnology companies are hesitant to invest in mAb research and development. We therefore need alternative financing models, such as advanced market commitments. To ensure that mAbs reach populations who need them the most, the WHO prequalification programme could facilitate the establishment of procurement mechanisms.

Finally, the use of approved mAbs products for persistent infections and/or mutating pathogens is of concern. As with other drugs, antimicrobial resistance to mAbs is a potential threat. However, this threat may be overcome by targeting highly conserved epitopes or by using antibody cocktails containing more than one mAb. Postmarketing guidelines to monitor mAbs efficacy would also be required.

In conclusion, mAbs have a potential to address a wide range of infectious diseases and development pathways need to be clearly defined to facilitate the licensure of these products. Regulatory agencies, biotechnology companies, public sector research agencies and funders must together implement a multisectoral approach to ensure adequate financing, clear regulatory guidance and policies to support the development, approval and use of mAbs. Such an approach extends beyond mAbs to other therapeutic products with difficult clinical pathways and uncertain markets. Global strategies, such as the WHO research and development Blueprint^18^ and other initiatives, will be essential for product development progresses.
